# Outcome of Delayed Administration of Alteplase in a Resource-Poor Area: A Case Report

**DOI:** 10.7759/cureus.25996

**Published:** 2022-06-16

**Authors:** Ahmed O Idowu, Ahmad A Sanusi, Simon A Balogun, Christopher O Anele, Akintunde A Adebowale, Abdulmajeed K Abidoye, Gloria J Akinola, Michael B Fawale, Morenikeji A Komolafe

**Affiliations:** 1 Department of Internal Medicine, Neurology Unit, Obafemi Awolowo University Teaching Hospitals Complex, Ife, NGA; 2 Department of Surgery, Neurosurgery Unit, Obafemi Awolowo University Teaching Hospitals Complex, Ife, NGA; 3 Department of Radiology, Obafemi Awolowo University Teaching Hospitals Complex, Ife, NGA

**Keywords:** antiplatelets, nigeria, resource-poor setting, ischemic stroke, thrombolytic

## Abstract

An acute ischemic stroke, though carrying the risk of debilitating complications, is a preventable and treatable disease. Thrombolysis and endovascular thrombectomy are important components of its management. However, various challenges in resource-poor countries like Nigeria and other developing nations pose a great limitation in the timely intervention of ischemic stroke treatment. The challenges include late presentation, poor awareness of stroke symptoms even among health care workers, poor ambulance service/transportation network, intra-hospital delay, particularly in neuroimaging, and the unavailability of tissue plasminogen activator (alteplase/tenecteplase).

We report a 32-year-old African man with an antecedent history of suspected migraine headaches with aura and a family history of hypertension and stroke, admitted 7½ hours after onset of stroke symptoms, scoring 13 on the National Institutes of Health Stroke Scale (NIHSS) with Medical Research Council (MRC) muscle power grades 1 and 3 on the right upper and lower extremities, respectively. Urgent non-contrast brain CT revealed only a hyperdense sign in the left middle cerebral artery (MCA). Intravenous tissue plasminogen activator (tPA) was administered at a lower dose of 0.6 mg/kg, 15½ hours after symptom onset, and a CT angiogram done 24 hours post-thrombolysis showed partial recanalization of the M1 segment of the MCA and intermediate collateral supply (Alberta stroke program early CT {ASPECT} score: 6). By the third day of admission, he had made a significant clinical improvement and was discharged home able to walk unsupported on the fourth day.

## Introduction

Stroke remains a leading cause of long-term disability and the second most common cause of death worldwide after ischemic heart disease [1-4]. It accounts for almost 5% of all disability-adjusted life-years and 10% of all deaths worldwide with the bulk of this burden occurring in low-income and middle-income countries of the world [5]. Acute ischemic strokes constitute about 80-85% of all strokes and thrombolytic therapy has been in use in its management for over two decades [6]. It is recommended that thrombolytic therapy be administered within a period of 4½ hours after the last known well (LKW) time or time of symptom onset [7]. Intravenous recombinant tissue plasminogen activator (rt-PA) is usually given at a dose of 0.9 mg/kg within this window. However, there is little evidence of the use of thrombolytics with a favorable outcome when the window period of intervention has been far exceeded [8].

This limited window of the proven benefit of thrombolytic therapy in addition to the unavailability and high cost of thrombolytics has created a challenge for patients in the African setting where the time from LKW to presentation is often significantly longer, thus excluding most patients from thrombolytic therapy. Poor recognition of stroke symptoms even among health care workers, late hospital presentation, intra-hospital delay particularly in neuroimaging, and lack of availability of well-equipped stroke units in most tertiary centers in the country also contribute significantly to the poor outcome in most patients with ischemic stroke. Here, we presented a case of acute ischemic stroke due to occlusion in the middle cerebral artery with the use of thrombolytic therapy well beyond the window period, albeit at a reduced dose, and with an excellent clinical outcome.

## Case presentation

A right-handed 32-year-old male, known migraineur was admitted to the emergency department on account of 7½ hours history of headache, 2½ hours of confused speech, and sudden onset of right-sided body weakness of 30 minutes, before the presentation. The headache was sudden in onset, quite similar to his usual migraine headaches, and was minimally relieved by the intake of caffeine/ergotamine tablet. He subsequently noticed intermittent confusion in his speech 2½ hours before presentation while conversing with his friends and decided to take a nap.

He however woke up 2 hours later with a sudden onset of right-sided body weakness, maximal at onset, affecting the right upper limb more than the right lower limb with associated slurring of speech and facial deviation to the left. There was no report of loss of consciousness, seizures, sphincteric incontinence, vertigo, vomiting, and no antecedent history of fever, neck pain, or neck stiffness. His last meal was 6 hours before the presentation.

He had a background history of suspected migraine headaches with aura described as zigzag lines in his vision with no history of any other chronic medical illness and denied intake of alcohol, smoking, or use of illicit drugs. His only medication was caffeine/ergotamine tablets which he used one to two times per week for his migraine headache attacks. He had a family history of hypertension and stroke in his first-degree relatives.

Examination vitals revealed a temperature of 36.90°C, respiratory rate of 18 cycles per minute (cpm), bradycardia (pulse rate: 54 beats/minute, regular), and blood pressure of 134/64 mmHg. On neurological examination, he was conscious, slightly restless with expressive aphasia, central peripheral facial palsy (upper motor neuron palsy), right flaccid hemiparesis with Medical Research Council (MRC) muscle power grade 1 in the right upper limb and 3 in the right lower limb, and absent plantar response on the right with no sensory or cerebellar deficits.

**Figure 1:**
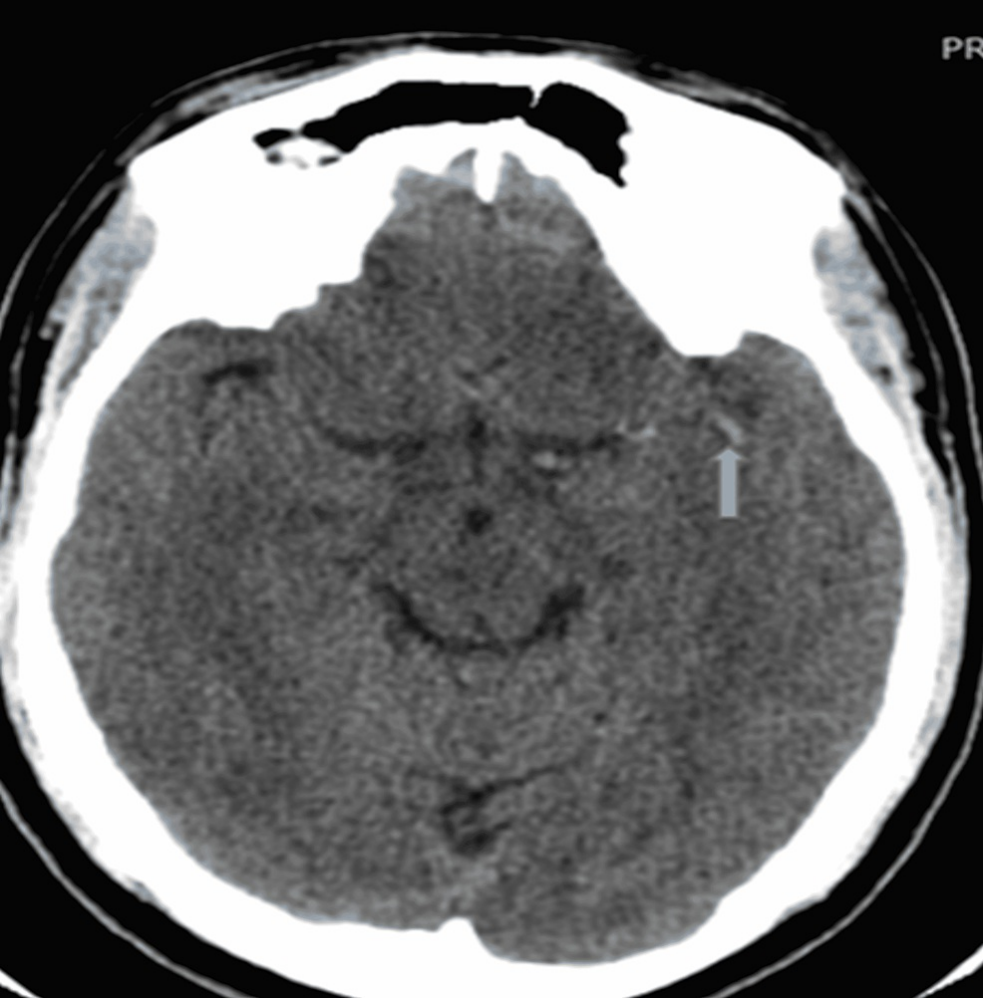
Linear hyperdensity in the region of the left MCA consistent with dense MCA sign of hyperacute infarct. MCA: middle cerebral artery

**Figure 2:**
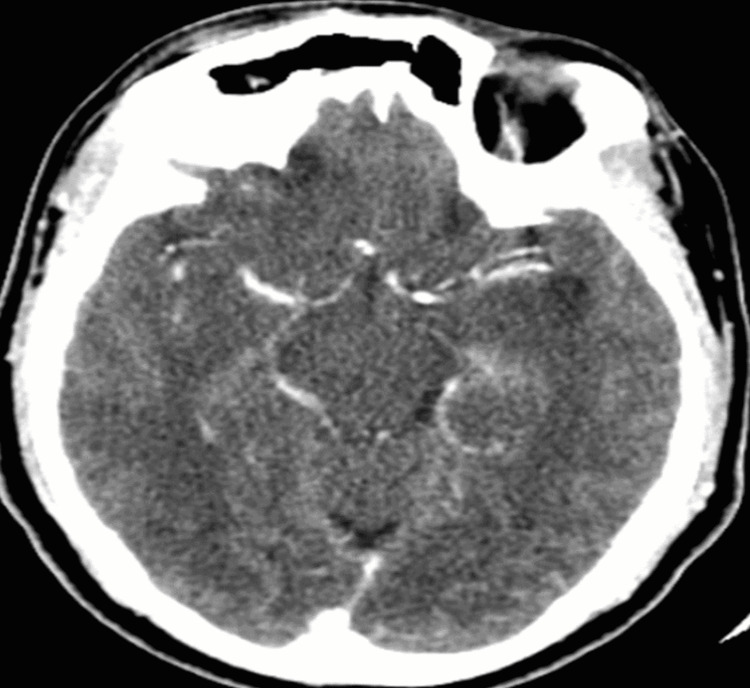
CT angiogram showing opacification of the left MCA post-administration of alteplase. MCA: middle cerebral artery

**Figure 3:**
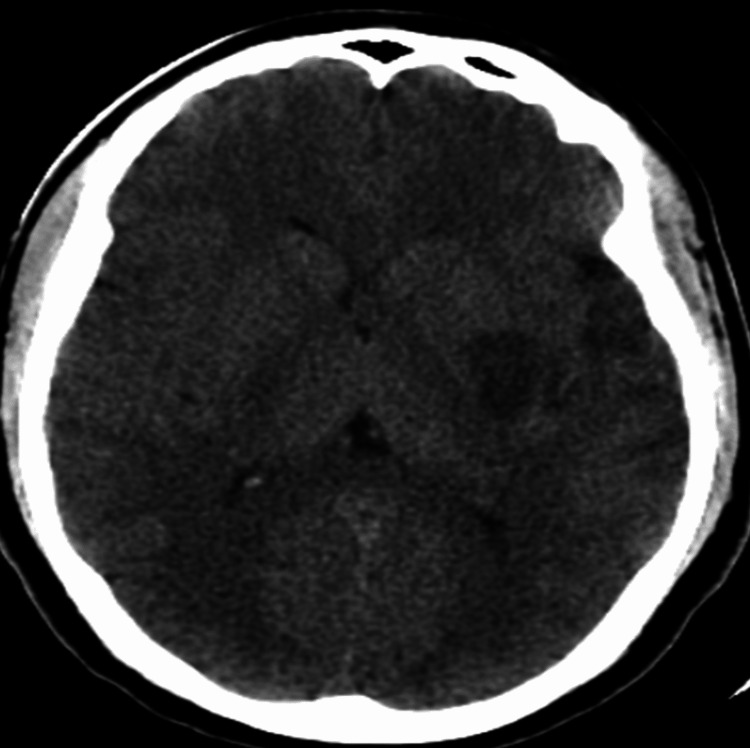
Post-alteplase hypodensities involving the left basal ganglia and left Sylvian fissure (left MCA territory). MCA: middle cerebral artery

Further cardiovascular examination revealed a grade 2 apical and left parasternal pansystolic murmur on auscultation, with no audible carotid bruit. He was evaluated as a case of acute left hemispheric ischemic stroke in the middle cerebral artery territory (National Institutes of Health Stroke Scale {NIHSS} score - 13 and modified Rankin Scale {mRS} - 4). Investigations were carried out and he was admitted directly into the ICU for close monitoring. The investigation results are shown in Table 1 and Figures 1-3 below.

**Table 1:** Summary of results of investigations. MCA: middle cerebral artery; RBG: random blood glucose; FBC: full blood count; COIVD-19: coronavirus disease 2019; PCR: polymerase chain reaction; INR: international normalized ratio; PT: prothrombin time; PTTK: partial thromboplastin time with kaolin; PCV: packed cell volume; LDL: low-density lipoprotein; HDL: high-density lipoproteins

Urgent non-contrast brain CT	Left hyperdense MCA sign
RBG	4.6 mmol/L
FBC	PCV: 51%, WBC: 6,400 cells/mL, neutrophils: 73%, lymphocytes: 26%, monocytes: 1%, platelets: 120,000/mL
Clotting profile	INR: 1.0, PT test: 14 s (control: 14 sec), PTTK test: 35.0 s (control: 35 s)
12 lead ECG	Normal findings
Lipid profile	Total cholesterol: 5.6 mmol/L, LDL: 4.1 mmol/L, HDL: 1.03 mmol/L, triglycerides: 1.1 mmol/L
Electrolytes/urea/creatinine	Cr: 106 µmol/L (50-132 µmol/L, K: 3 mmol/L (3-5 mmol/L), Cl: 103 mmol/L (90-110 mmol/L), Na: 136 mmol/L (135-145 mmol/L), urea: 3.9 mmol/L (2.5-5.8 mmol/L)
COVID-19 PCR	Negative
Transthoracic echocardiography	Normal pericardium, no pericardial effusion, normal cardiac valves and chambers, preserved left ventricular systolic and diastolic function, mild aortic, tricuspid, and mitral regurgitation

Immediate thrombolytic therapy was to be commenced; however, it was not available in the hospital or any pharmacy in the city where the hospital is located. Alteplase was also unavailable in nearby states and the availability was eventually confirmed in a larger city about 400 km away. The drug was purchased and had to be transported by road, the only available means between the two cities.

Eight hours into the intensive care unit (ICU) admission (15½ hours post-stroke), 50 mg IV alteplase was administered at a lower dose of 0.6 mg/kg - 10% (5 mg) given as a bolus over 1 minute, and the remaining 90% (45 mg) added to 400 mL of 9% normal saline (N/S) to run over 1 hour and commenced on physiotherapy. The decision to administer the alteplase at such a low-dose at the time period was based on the discretion of the multidisciplinary team led by the consultant neurologists.

The vital signs were monitored closely, and he had a repeat brain CT+CT angiography (24 hours after alteplase administration) which showed a hypodense region in the left temporal lobe with partial recanalization of the M1 segment of the middle cerebral artery and good vascular collaterals (Alberta stroke program early CT {ASPECT} score - 6) (Figures 2, 3).

On the third day of admission, he had made a remarkable improvement with a power of four in the right upper limb and lower limb and vocalizing with appropriate answers to questions. Reading, naming, and repetition were intact but had minimal difficulty comprehending a three-stage command. He was discharged home with an mRS of 1 and able to walk unsupported on the fourth day on oral atorvastatin and low-dose aspirin and counseled to continue outpatient physiotherapy and follow-up in the neurology clinic.

## Discussion

The management of acute ischemic stroke requires a timely intervention with the use of antiplatelets, intravenous thrombolytics, and endovascular/mechanical thrombectomy [9]. Delay in presentation or initiation of treatment tends to produce adverse clinical outcomes.

Most studies have demonstrated remarkable clinical outcomes with thrombolysis within 3-4.5 hours of reperfusion in acute ischemic stroke. Hacke et al. observed improved clinical outcomes in those who received IV alteplase within 3-4.5 hours, and Saver et al. noted a reduction in mortality and the risk of symptomatic intracranial hemorrhage when administered early [7,9]. Recent advances in endoscopic intervention with mechanical thrombectomy have also proved useful in large vessel occlusion within 6-24 hours after the last known well (LKW) time in the absence of contraindications and good pre-functional status [10].

The index case presented with a left middle cerebral artery (MCA) occlusion confirmed on non-contrast brain CT and was managed with intravenous rt-PA (15.5 hours after onset of symptoms) with a desirable outcome (Figure 1). This suggests the need for further research on the role of delayed thrombolysis while balancing the risks and benefits in resource-poor settings like Africa where rt-PA is not readily available, accessible, or affordable. Studies such as Extending the Time for Thrombolysis in Emergency Neurological Deficits (EXTEND), European Cooperative Acute Stroke Study 4 (ECASS4)-EXTEND, and Echo-Planar Imaging Thrombolytic Evaluation Trial (EPITHET) revealed the effectiveness of delayed thrombolytic intervention.

Current recommendations state that IV thrombolysis should be initiated in acute ischemic stroke within 4.5 hours after LKW time if there are no contraindications. There is a high chance of hemorrhagic conversion of the infarct with every hour delayed in thrombolysis and the ideal door-to-needle time has been estimated to be an hour. However, the index patient otherwise eligible for IV thrombolysis had far exceeded the window period at the time of brain CT acquisition and the unavailability of rt-PA within the hospital facility complicated the delay in thrombolysis. Based on reported cases of the use of lower doses of rt-PA, than recommended, with good response and a reduced risk of intracerebral hemorrhage, the team's decision was made to cautiously administer the IV rt-PA at a reduced dose (0.6 mg/kg) despite the attending risks and possible complications [11]. Evidence from a previous randomized controlled trial revealed that patients with acute ischemic stroke did not show the non-inferiority of low-dose alteplase to standard-dose alteplase with respect to death and disability at 90 days, [11] while other studies showed that low-dose and high-dose IV alteplase are comparable in effectiveness [12,13]. In addition, there were significantly fewer symptomatic intracerebral hemorrhages and reduced mortality among elderly patients, with low-dose alteplase [13]. However, further investigation is still being warranted to identify patients with acute ischemic stroke (AIS) who may benefit from low-dose alteplase. Perhaps, individuals presenting outside the recommended window period may benefit from low-dose alteplase as in our index case.

Meta-analyses of the EXTEND, ECASS4-EXTEND, and EPITHET studies provide evidence supporting delayed thrombolytic intervention in patients with favorable perfusion imaging (CT perfusion or perfusion-diffusion MRI) up to 9 hours after an ischemic stroke with remarkable outcomes [14-16]. Our patient had an IV thrombolytic administered 15.5 hours after the onset of symptoms and had an improved neurological outcome. This brings to the fore the relevance of continuously engaging in further research, especially among blacks, on what exact duration might administration of thrombolytics be deleterious in acute ischemic stroke, especially in settings with poor access to thrombolytics and late presentation to the hospital.

## Conclusions

Our case highlighted the challenges faced in ensuring a favorable outcome in acute ischemic stroke in resource-poor settings where recombinant tissue plasminogen activator is very expensive and not readily available. Furthermore, late presentation, poor awareness, and recognition of stroke symptoms in the community and among health care workers, the intra-hospital delay also contribute to the morbidity and mortality associated with ischemic stroke management.

We recommend certain initiatives such as a subsidized or funded “acute ischemic stroke package,” to include brain CT scan and thrombolysis in a single fee, a proper insurance health package that can cover the aforementioned, and increased sensitization and awareness programs on stroke in the community, the recognition of the Facial drooping, Arm weakness, Speech difficulties and Time (FAST) symptoms of stroke, and more policies and advocacy by policymakers with the aim of achieving a sustainable global reduction in the morbidity and mortality attributed to stroke.
